# Synthesis and Characterization of Nano‐Crystallite Triple Super Phosphate (TSP) from Marine Mollusk Waste: *Babylonia japonica*, *Oliva sayana*, *and Conasprella bermudensis*

**DOI:** 10.1002/open.202400174

**Published:** 2024-09-09

**Authors:** Md. Kawsar, Md. Sahadat Hossain, Sumaiya Akter, Md. Farhad Ali, Newaz Mohammed Bahadur, Samina Ahmed

**Affiliations:** ^1^ Glass Research Division Institute of Glass & Ceramic Research and Testing Bangladesh Council of Scientific and Industrial Research (BCSIR) Dhaka 1205 Bangladesh; ^2^ Department of Applied Chemistry and Chemical Engineering Noakhali Science and Technology University Noakhali Bangladesh; ^3^ Institute of Leather Engineering and Technology University of Dhaka Dhaka 1000 Bangladesh

## Abstract

The study aims to synthesize nano‐crystallite TSP using renewable, low‐cost, waste marine mollusk from three different species such as Babylonia japonica, Oliva sayana, and Conasprella bermudensis. The molar ratio of phosphate to calcium in triple superphosphate [TSP, Ca(H_2_PO_4_)_2_.H_2_O] significantly impacts its properties and fertilizer performance, in this case, we kept the ratio to 2. Raw TSP has a high phosphate content and lower calcium content. The synthesized TSP was analyzed using various techniques including TGA, XRD, EDX, FT‐IR, and SEM. The study utilized multiple XRD model equations to analyze crystallite size (<100nm ), with all models except the Liner straight‐line method providing higher estimates for synthesized TSP. Furthermore, the values for stress (2×10^7^ to 4×10^7^ N/m^2^), strain (4×10^−4^ to 9×10^−4^), as well as energy density (4.54×10^3^ to 16.27×10^3^ J/m^3^) were also calculated for the synthesized product. However, the preferential growth calculation indicates that (010), (021), and (020) planes are the most thermodynamically stable planes for the growth of the synthesized TSP. Apart from that, FTIR result confirms that CaO, −OH, as well as PO_4_^3−^ functional groups are present in the synthesized products. This research suggests that marine mollusks can be utilized as a calcium precursor for P‐fertilizer and 60 % phosphoric acid, thereby reducing production costs by eliminating additional dehydrating. Additionally, waste marine mollusk shells could be utilized as an alternative to the production of phosphate‐based fertilizer.

## Graphical abstract

The study explores the synthesis of nano‐crystallite triple super phosphate (TSP) from marine mollusk waste, and in‐depth crystallographic characterization carries good evidence for fruitful application as nano‐fertilizer.

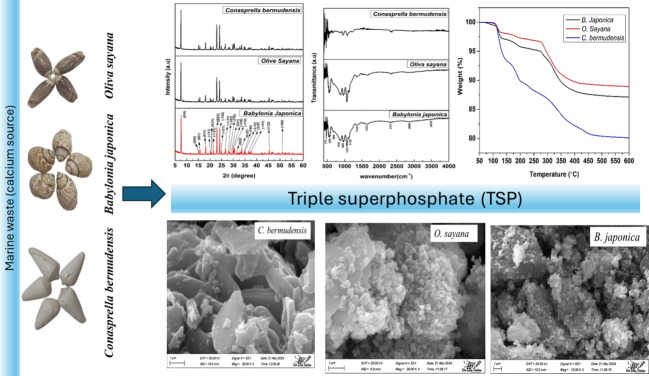

## Introduction

The growing global population necessitates an increase in food production, but the issue is exacerbated by a decrease in agricultural land availability.^[1]^ Experts worldwide are focusing on enhancing food production by utilizing limited land resources. Chemical fertilizers, primarily nitrogen, phosphorus, and sulfur‐based, are being used to boost crop yield, providing significant benefits.^[2]^ Phosphate‐based fertilizers are mostly derived from rock phosphate minerals, which are becoming diminished in availability.[^3^, ^4^] Research suggests that by 2035, the demand for phosphorus is expected to surpass its availability, potentially leading to its eventual elimination.^[3]^ Phosphorus‐based fertilizer demand has surged from 5 million MT in 1961 to 18 million MT in 2018, projected to reach 22 to 27.2 million MT by 2050.^[5]^ A study in Nature Proceedings revealed that globally, approximately 140 million tons of rock phosphates are generated as well as utilized, with the majority used for phosphate fertilizer production.^[6]^ Some countries are worried about the lack of phosphate minerals enabling the manufacturing of P‐fertilizer.^[7]^ Researchers are focusing on finding phosphorite concentrates efficiently and sustainably, despite rock phosphate being used as a conventional supply for P‐fertilizer synthesis.^[8]^ The traditional industrial process for manufacturing superphosphate is time‐consuming due to the reaction between rock phosphate and sulfuric acid.^[9]^ The purity of phosphate‐based fertilizer, which is around 90 %, is causing issues in its application and industrial efficiency.^[10]^ The World Bank predicts a global increase in municipal solid waste, including bio‐waste, to 2.2 billion tons annually by 2025, with a twofold increase in developing countries.^[11]^ The study of bio‐waste disposal, including its exploitation and recycling for sustainable development, has gained significant attention.[^12^, ^13^, ^14^] TSP production traditionally uses non‐renewable phosphate rock ore, leading to higher costs. However, renewable precursors like *Babylonia japonica*, *Oliva sayana*, and *Conasprella bermudensis* have significantly reduced manufacturing costs, resulting in lower calcium carbonate costs compared to phosphate rock ore.[^15^, ^16^] Biomass‐derived renewable precursors, known as biomimetic precipitation, are used to provide calcium and phosphate, the primary components of TSP.^[17]^ The transition towards renewable precursors has reduced environmental toxicity and safeguarded natural resources like limestone and phosphate rock ore.^[18]^ Renewable precursors significantly impact TSP precipitation by providing calcium and phosphate through biomimetic precipitation, forming nanosized calcium phosphate precursors. These precursors are more effective in reducing soil pH and promoting plant growth compared to traditional TSP production methods. Additionally, renewable precursors enable the production of TSP with specific properties, such as improved biocompatibility and biocorrosion resistance.^[17]^ Apart from that, TSP uses *Trichoderma spp*. to enhance soil phosphate solubility, generate slow‐release fertilizers, and analyze maize hybrid breeding under reduced P starting fertilizer. Monetite from brushite is another option, but phosphate rock‘s purity and mineral resource limitations limit its use.^[19]^ The research demonstrates the use of renewable marine mollusk waste as precursors for TSP synthesis, a significant advancement compared to traditional reliance on non‐renewable phosphate rock ore. The shift towards renewable precursors promotes a more sustainable production process for TSP. The research contributes to the development of more environmentally friendly and resource‐efficient fertilizers and materials, offering a promising alternative to traditional TSP production methods.

## Materials and Methods

### Materials

The key raw materials of the study are *Babylonia Japonica, Olive Sayana, and Conasprella bermudensis*, which were collected from Cox's bazar region. Once the mollusk shells were collected, they were properly cleaned with tap water and then left to dry in the sun. Concentrated (85 %) orthophosphoric acid for the synthesis process was purchased and shipped from E‐Merck, Germany, which was used directly without any further treatment. The reactants for this process also include deionized water, which was amassed from Glass Research Division, IGCRT, BCSIR, the same site where it was prepared.

#### Synthesis of Triple Superphosphate

This study focuses on the production of TSP using waste mollusk shells from three species, *Babylonia Japonica*, *Olive Sayana*, and *Conasprella bermudensis*, as a calcium source. This differs from previous research that used phosphate rock ore or pure calcium carbonate as the precursor. The three shell types were used individually to prepare separate TSP products, unlike previous works that likely used a single calcium precursor. The synthesis process is straightforward, involving a direct reaction of diluted phosphoric acid with each shell‐derived CaCO_3_ powder, compared to more complex methods used in previous studies. To carry out the synthesis process of TSP at a lab scale, a batch mode protocol was adopted. At the beginning of the process the reactants, orthophosphoric acid and calcium carbonate (shells of the three mentioned mollusk), were prepared to facilitate the reaction. The 85 % (w/w) orthophosphoric acid was mixed with adequate amount of deionized water in a vessel to reduce the acid concentration to 60 % (w/w). The dried shells of *B. japonica, O. Sayana*, *and C. bermudensis* were ground into a fine powder using a high‐speed ball mill to improve reaction rate. The acid and powdered shells were mixed by agitation, and the powder to acid molar ratio was regulated at 1 : 2. The mixture was agitated until the reaction between orthophosphoric acid and calcium carbonate was complete, as illustrated in the equation 1

(1) $${CaCO}_{3}\left(Powdered\;shells\right)+H_{3}{PO}_{4}\;+H_{2}O\to {Ca\left(H_{2}{PO}_{4}\right)}_{2}.H_{2}O+Heat$$

The process involved trapping 1 mole of water within a TSP crystal, which was removed by heat during the hydration reaction, without filtration or heating. The synthesized TSP was dried at ambient conditions within 24 h and stored for further analysis.

### Characterization

#### Thermogravimetric Analysis (TGA)

The thermal characteristics of synthesized products were assessed using a simultaneous thermal analysis equipment, with a temperature range of 50 to 600 °C and a heating rate of 20 °C per minute, using an inert environment with the aid of nitrogen gas.

#### X‐Ray Diffraction Analysis

The powdered sample underwent crystallographic phase analysis using a Rigaku Smart Lab XRD machine. The machine was operated at 40 kV and 50 mA, with a tubular wire radiation source. The XRD pattern was recorded with 2θ ranging from 5° to 60°, maintaining temperature at 23 °C and cooling water flow rate of 4.6–4.8 L min^−1^. Data were recorded using the Bragg‐Brentano para‐focusing geometry mode, which was also calibrated against a silicon standard.

#### FT‐IR Spectroscopic Analysis

The sample‘s functional groups were identified through FT‐IR spectroscopic analysis using an IR‐Prestige 21 machine with an attenuated total reflection system. The data were collected by maintaining the transmittance mode in the range of 400 to 4000 cm^−1^ and maintaining a spectral resolution of 4 cm^−1^. The visualized data were an average of 30 scans.

#### SEM Analysis

The morphological analysis of synthesized TSP was conducted using a FESEM instrument, specifically the JEOL JSM‐7610F, at a 15 kV accelerating voltage.

## Results and Discussion

### Thermogravimetric Analysis (TGA)

**Figure 1:**
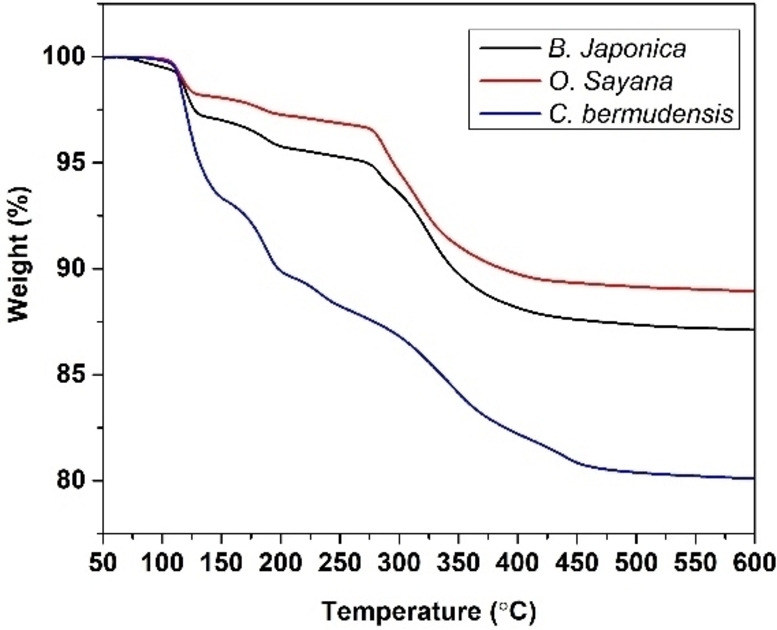
TGA analysis of synthesized TSP.

A thermogravimetric analysis (TGA) study on synthesized TSP samples (Figure 1) revealed their thermal behavior and breakdown properties after a gradual temperature increase from 50 °C–600 °C under a controlled atmosphere. The researchers observed weight reduction in TSP samples, revealing composition, stability, and breakdown kinetics. The weight loss was likely owing to water removal and the breakdown of the calcium dihydrogen phosphate molecule in the TSP. Mass loss below 100 °C was due to free moisture removal, resulting in 0.38 % moisture evaporation. Three phases of dehydration mechanisms were identified in the TSP crystal structure: the first phase involved water removal, the second involved the synthesis of calcium dihydrogen pyrophosphate, and the third, over 350 °C, resulted in water release, causing a near 8 % mass loss. The TGA features of the synthesized TSP is given in Table 1.

**Table 1:** TGA features of synthesized TSP.

**Synthesized TSP**	**Temperature range (°C)**	**Weight loss (%)**
**B. Japonica**	109–160	2.81
	160–259	1.79
	259–600	8
**O. Sayana**	102–126	1.73
	126–273	1.37
	273–600	7.87
**C. bermudensis**	104–147	6.48
	147–202	3.78
	202–370	6.16
	370–600	3.03

### Crystallographic Analysis

**Figure 2:**
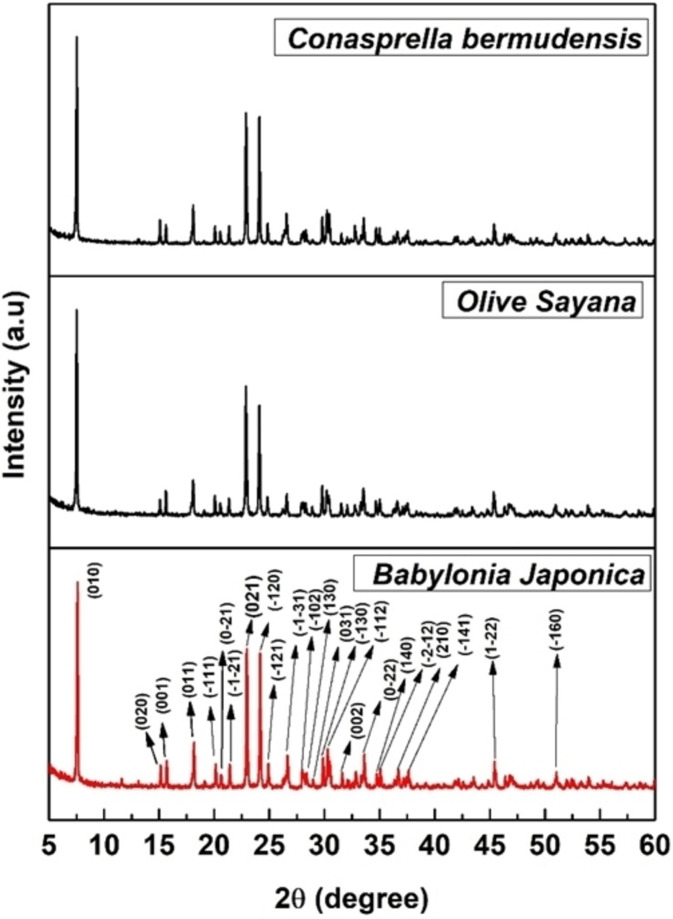
X‐ray diffraction pattern triple super phosphate.

The product‘s crystallographic study was conducted using powder X‐ray diffraction data, as depicted in Figure 2, which includes a diffractogram and accompanying planes. The synthesized TSP′s XRD profile closely matched the standard ICDD reference (card no.# 04‐011‐3010).

The observation validated the phase of the generated product as CaP‐based TSP. The corresponding 2θ locations along with the planes of the sample are 7.61 (010), 15.02 (020), 15.72 (001), 18.18 (011), 20.14 (−111), 20.64 (0‐21), 21.49 ( −1‐21), 22.91 (021), 24.25 (−120),25.00 (−121) 26.65 (−1‐31), 28.00 (−102), 28.36 (130), 28.96 (031), 29.86 (−130), 30.32 (−112), 31.67 (002), 33.62 (0‐22), 34.77 (140), 35.13(−2‐12), 36.79 (210), 37.69 (−141), 45.41 (1‐22), 51.08 (−160). The data confirms the creation of a single phase of TSP, as all exhibited reflections align with TSP criteria and no another phase was documented. The crystallographic characteristics, like size of the crystallite, microstrain, crystallinity index, dislocation density as well as specific surface area were determined using eq^n^ (2)‐(6), and the details of these equations are discussed elsewhere.[^20^, ^21^] The estimated crystallographic variable of synthesized TSP is given in Table 2.

(2) Crystallitedimension,Dc=kλ/βCosθ

(3) Microstrain,ϵ=β/4tanθ

(4) $$\mathrm{Crystallinity}\;\mathrm{index},\;{CI}_{\mathrm{XRD}\;}=\sum \frac{H_{\left(010\right)}+H_{\left(021\right)}+H_{\left(-120\right)}}{H_{\left(010\right)}}$$

(5) $$\mathrm{Dislocation}\;\mathrm{density},\;\delta =\frac{1}{{\left(D_{c}\right)}^{2}}$$

(6)

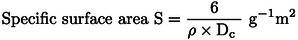




**Table 2:** Crystallographic variables of synthesized TSP.

Parameter	*B. Japonica*	*O. Sayana*	*C. bermudensis*
Crystallite size, Dc	113.739	99.51	88.06
Microstrain, ϵ	4.60× 10^−3^	5.30× 10^−3^	6.00× 10^−3^
Crystallinity index, CI_XRD_	2.58	2.48	2.31
Dislocation density, δ (10^15^ lines per m^2^)	0.077	0.10	0.12
Specific surface area, S(m^2^g^−1^)	0.023	0.027	0.030

Preference growth (PI) (equation 7) is a crucial characteristic for crystallographic characterization of materials, where the preference of a crystal plane increases under specific experimental conditions, calculated by matching the relative intensity (RI) (equation 8) of one plane to the different strong planes. To estimate RI of synthesized TSP, (010), (021), (−120), as well as (020) planes are taken into consideration.

(7) $$P=\;\frac{RI-{RI}_{s}}{{RI}_{S}}$$

Here, RI and RIS denotes relative intensity of the sample and relative intensity of the standard, correspondingly.

(8) $$\mathrm{Relative}\;\mathrm{intensity},\;\mathrm{RI}=\;\frac{I_{\left(010\right)}}{I_{\left(021\right)}+I_{\left(-120\right)}+I_{\left(020\right)}}\;$$

These findings clearly suggested that the preferential growing along (021), (020) for *B. Japonica*, and (010), (021), (020) for *O. Sayana*. Furthermore, (010), (021), (020) for *C. bermudensis*. These planes in the synthesis of TSP showed a thermodynamically favorable preference, while negative signals indicated a decreasing preference throughout the reaction within the prescribed limits. Table 3 shows the values of preference growth and relative intensity of the synthesized products.

**Table 3:** Relative intensities and Preference growth of synthesized TSP.

Synthesized TSP	Considered Plane	Intensities of the plane	RI of the sample	Intensity of the reference planes	RI_s_ intensity	Preferential growth
B. Japonica	010	100	0.57	100	0.58	‐0.01
	021	89.13	0.48	82.3	0.43	0.10
	‐120	74.64	0.37	80.2	0.42	‐0.11
	020	9.04	0.03	7	0.02	0.28
O. Sayana	010	100	0.59	100	0.58	0.008
	021	88.33	0.49	82.3	0.30	0.60
	‐120	71.42	0.36	80.2	0.42	‐0.142
	020	8.35	0.032	7	0.02	0.20
C. bermudensis	010	100	0.59	100	0.58	0.011
	021	83.92	0.45	82.3	0.43	0.03
	‐120	72.4	0.37	80.2	0.42	‐0.12
	020	11.29	0.04	7	0.02	0.65

### Crystallite Size Estimation by Implementing Various Models

#### Scherrer Model

The Scherrer equation is a widely used model for estimating crystallite size from X‐ray diffraction analysis peak broadening. It establishes mathematical relations between Ds, K, λ, β, and θ, representing crystallite size, shape factor, X‐ray source wavelength, full width at half maxima, and Bragg's angle. K is a constant with a value of 0.90. This model can be written as equation 9.[^22^, ^23^, ^24^, ^25^, ^26^]

(9) $$D_{s\;}=\frac{K\lambda }{\;\beta .cos\theta }$$

Often, while considering the data output from the XRD analysis, instrumental broadening is likely to incorporate the peak width. In such case the XRD data gives a less accurate output where the value of peak broadening is deviated from the actual value resulting an error. To eliminate this error, the instrumental broadening is computed by running a standard sample and then comparing the result with the predetermined standard value for the sample. Then the computed value of instrumental broadening is used to deduce the value of actual full width at half maxima using equation 10.[^26^, ^27^, ^28^]

(10) $${\beta_{actual}}^{2}\;=\;{\beta_{measured}}^{2}-{\beta_{instrumental}}^{2}$$

(11) $$\beta_{\mathrm{actual}}=\;\sqrt{{\beta_{\mathrm{measured}}}^{2}-{\beta_{\mathrm{instrumental}}}^{2}}\;$$

The calculated value of crystallite size for the powdered TSP sample using equation (9) was estimated as 98.67, 81.88, and 95.12 nm for *B. Japonica, O. Sayana and C. bermudensis*, correspondingly.

#### Liner Straight‐Line Method of Scherrer Model (LSLMSE)

The Liner straight‐line method, a modified Scherrer method was adopted to calculate the size of crystallite more precisely considering all XRD peaks instead of a scattered specific peak. In this method, all peaks, or any selected scattering peak of the XRD plot can be studied. To develop this method, the Scherrer equation (equation (9)), was rearranged as shown in equation 12.[^29^, ^30^, ^31^]

(12) $$\cos\theta =\frac{K\lambda }{D_{c}}\times \frac{1}{\beta }=\frac{K\lambda }{D_{L}}\times \frac{1}{\beta }\;$$

**Figure 3:**
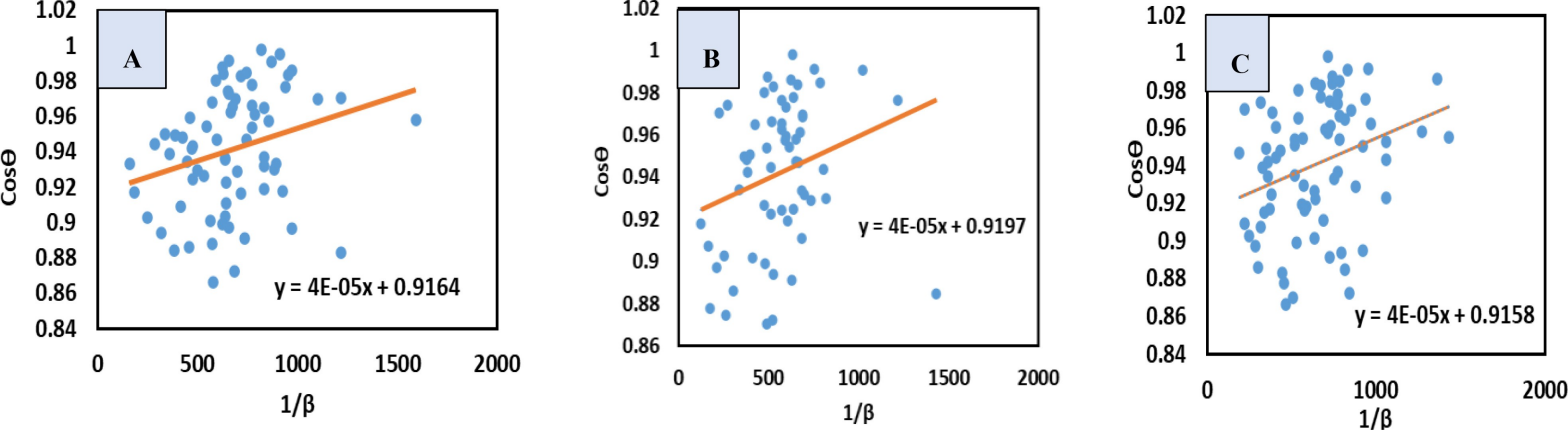
LSLM for (A) B. japonica (B) O. sayana (C) C. bermudensis.

This altered equation may be contrasted with the straight‐line equation, y=mx + c, where the slop is $\frac{K\lambda }{D_{L}}$ . A cosθ vs $\frac{1}{\beta }$ plot was drawn (Figure 3), and a straight line was generated from the plot. The equation obtained from the plot was compared with equation (12) to calculate the crystallite size. The crystallite dimension ($D_{c}$ ) of synthesized TSP was estimated as 3466.35, 3466.35, 3466.35 nm for *B. Japonica, O. Sayana and C. bermudensis*, respectively (Table 4).

**Table 4:** Different microstructural features TSP.

Model Name		Crystallite dimension (nm), stress (N/m^2^), energy density (J/m^3^)		
		*B. japonica*	*O. sayana*	*C. bermudensis*
Scherrer's equation		D_L_=98.67	81.88	95.12
Linear straight‐line method of Scherrer's method		D_L_=3466.35	3466.35	3466.35
Monshi‐Scherrer method		D_L_=104.87	91.21	101.68
Williamson‐Hall Method	UDM	ϵ =5×10^−4^ D_w_=126.04	ϵ =9×10^−4^ D_w_= 138.65	ϵ =4×10^−4^ D_w_= 115.54
	USDM	σ =2×10^7^ D_w_=126.04	σ =4×10^7^ D_w_= 138.65	σ =2×10^7^ D_w_= 115.54
	UDEM	u=4.82×10^3^ D_w_=126.04	u= 16.27×10^3^ D_w_= 138.65	u=4.54×10^3^ D_w_=115.54
Size‐strain plot		D_w_=106.65	81.56	86.65
Halder‐Wagner Method		D_w_=50	22.22	32.25
Sahadat‐Scherrer's Model		D_s‐s_=115.54	92.43	106.65

#### Monshi‐Scherrer Method (MSM)

The MSM, is a recently developed model to calculate crystallite size, eported to yield a precise result in numerous studies. It is deduced by rearranging the Scherrer equation and taking logarithm on both side of the equation. The model was designed to minimize mathematical errors when determining crystallite size using all peaks of a sample‘s XRD output. Adoption of logarithm made the calculation more specific and accurate. Equation (15) provides a mathematical representation of this model.

(13) $$D_{M}=\frac{K\lambda }{\beta cos\theta }$$

(14) $$\mathrm{Or},\;\beta =\frac{1}{\cos\theta }\times \frac{K\lambda }{D_{M}}\;$$

(15) $$\mathrm{Or},\;\ln\beta =\ln\frac{1}{\cos\theta }+\ln\frac{K\lambda }{D_{M}}\;$$

**Figure 4:**
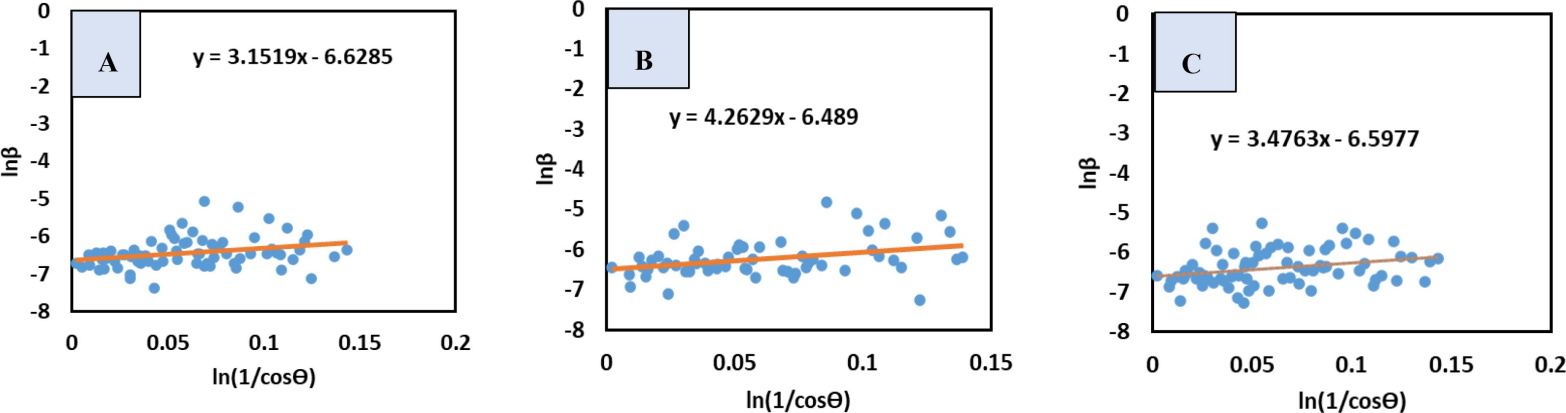
MSM for (A) B. japonica (B) O. sayana (C) C. bermudensis.

A graph was drawn placing lnβ (y‐axis) and $\frac{1}{\cos\theta }$ (x‐axis) (Figure 4), that generated a straight‐line equation which was lately compared with equation (15) to measure the crystallite size. The crystallite dimension ($D_{L}$ ) of synthesized TSP was estimated as 104.87, 91.21, and101.68 nm for *B. Japonica, O. Sayana and C. bermudensis*, correspondingly (Table 4).

#### Williamson‐Hall Plot

The formula developed by Scherrer is calculated by assuming that the peak widening of the XRD pattern is exclusively attributable to the size of the crystallite. But in fact, the peak broadening can also be affected by various intrinsic factors such as different kinds of defects, grain boundary, strain and so on. The Williamson‐Hall plot determines crystal size and strain in a crystalline material, using the combined influence of crystallite size as well as micro‐strain on XRD peak broadening. As these two factors affect the peak broadening, these certainly influence FWHM value. This concept of Williamson‐Hall plot is mathematically depicted by equation (16), where the total FWHM ($\beta_{\mathrm{total}})$ is the sum of FWHM resulted by the crystallite size and micro‐strain separately.[^36^, ^37^]

(16) $$\beta_{total}=\;\beta_{size}+\beta_{strain}$$

The variables, $\beta_{size}$ and $\beta_{strain}$ can be deduced from the Scherrer, Stokes and Wilson formula respectively. Here the Scherrer equation represents crystallite size (D), Stokes and Wilson formula represents the strain (ϵ) . These two expressions are shown in equation (17) and equation 19.[^34^, ^38^, ^39^]

(17) $$\beta_{size}=\frac{K\lambda }{D.\cos\theta }$$

(18) $$\epsilon =\frac{\beta_{strain}}{4\tan\theta }$$

(19) $$\mathrm{Or},\;\beta_{strain}=4.\;\epsilon .\tan\theta$$

Combining the two equations (17), and (18), yields equation 20.

(20) $$\beta_{total}=\frac{K\lambda }{Dcos\theta }+4.\epsilon .\frac{sin\theta }{cos\theta }$$

Equation (20) is the general mathematical expression of Williamson‐Hall model.[^40^, ^41^] The crystallite size and lattice strain of the sample were computed using the Williamson‐Hall approach employing three sub‐models: uniform deformation model (UDM), uniform stress deformation model (USDM), and uniform deformation energy density model (UDEDM).

### Uniform Deformation Model (UDM)

The UDM is taken into consideration when the intrinsic strain is oriented from isotopic deformation of crystals. The strain is uniform in all crystallographic directions in such circumstances. The equation regarding the UDM method can be written as equation (21) which is obtained by rearranging equation 20.[^37^, ^42^, ^43^]

(21) $$\beta_{total}\cos\theta =\;\frac{K_{B}\lambda }{D_{w}\;}+4\;\epsilon \;sin\theta$$

**Figure 5:**
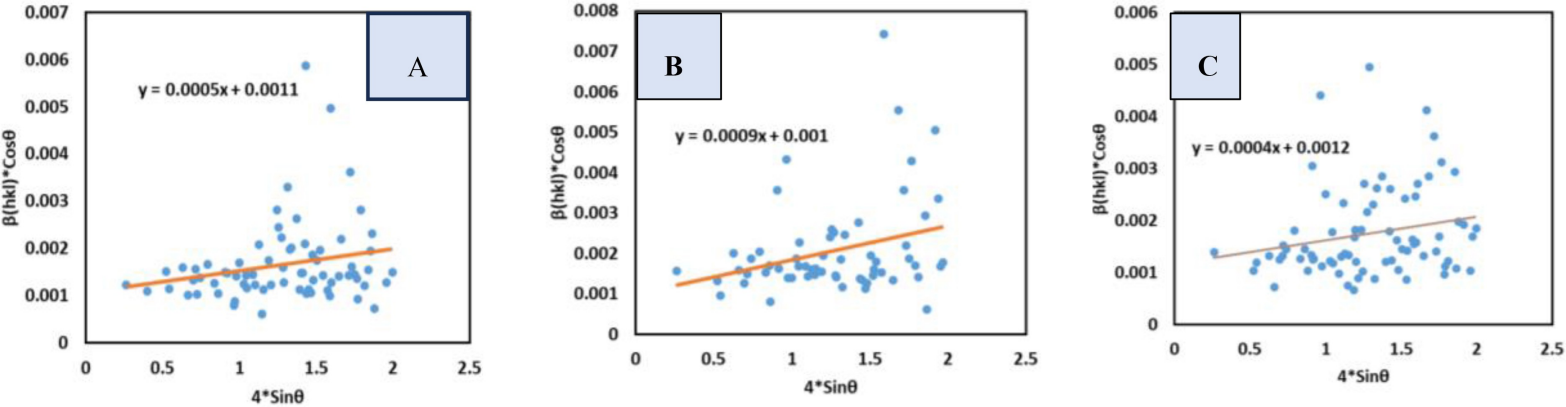
UDM for (A) B. japonica (B) O. sayana (C) C. bermudensis.

To measure the size of the crystallite as well as strain $\beta_{total}\cos\theta$ was plotted along the y‐axis, 4sinθ was plotted along the x‐axis and the resulting straight‐line equation was compared with the y=mx+c equation (Figure 5). The crystallite dimension was computed from the intercept and strain was computed from the slop. For *B. Japonica*, *O. Sayana*, and *C. bermudensis*, the crystallite diameter (D_W_) and strain of the synthesized TSP were found to be 126.04, 138.65, and 115.54 nm, respectively, and 5×10^−4^, 9×10^−4^, and 4×10^−4^, respectively, as shown in Table 4. According to this concept, a slop that is positive indicates that the crystal is undergoing tensile mode, whereas a slop that is negative suggests that the crystal is undergoing compressive strain.

### Uniform Stress Deformation Model (USDM)

The USDM model predicts that crystals are less likely to produce uniform strain deformation, since stress is consistently distributed along the crystallographic orientations in the lattice of the crystallites, connected with strain. Hook's law, as indicated in equation (22), establishes the link between stress and strain.[^44^, ^45^, ^46^]

(22) $$\sigma =Y_{hkl}.\epsilon \;$$

(23) $$\mathrm{Or},\;\epsilon =\;\frac{\sigma }{Y_{hkl}}$$

In this equation σ denotes to strain and $Y_{\mathrm{hkl}}$ denotes to young's modulus. By putting the value of ϵ in equation (20), the following equation (equation 24)) is found which is known as the USDM.[^21^, ^47^, ^48^]

(24) $$\beta_{total}\cos\theta =\;\frac{K_{B}\lambda }{D_{w}\;}+4\;\sigma \frac{\;sin\theta }{Y_{hkl}}$$

**Figure 6:**
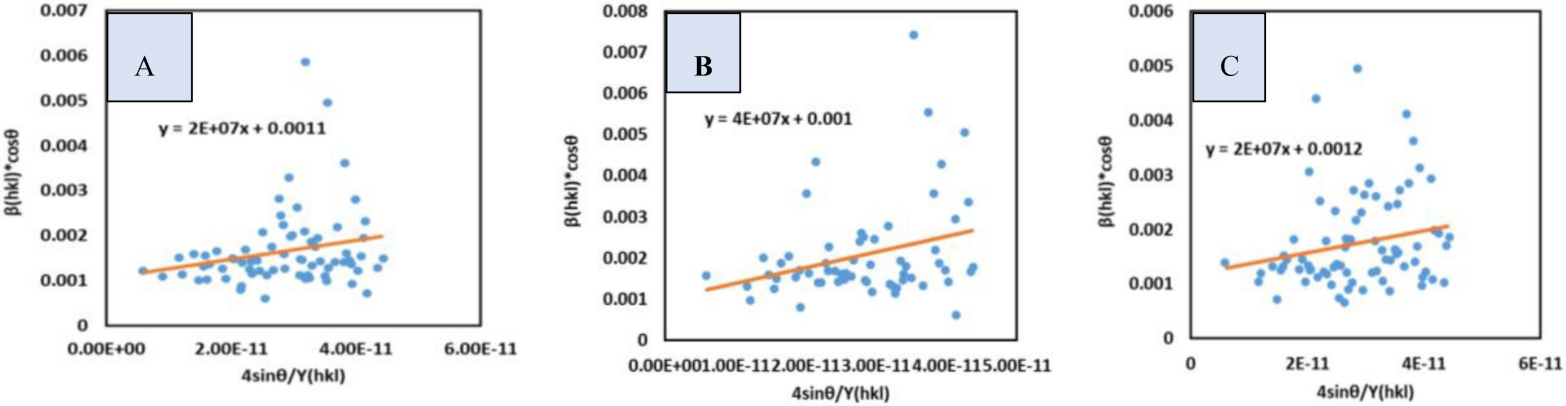
USDM for (A) B. japonica (B) O. sayana (C) C. bermudensis.

In this study the elastic modulus was considered as 45 Gpa.^[49]^ The calculation of size and stress were carried out by drawing a $\beta_{total}\cos\theta$ (vertical axis) vs $4\;\frac{\;sin\theta }{Y_{hkl}}$ (horizontal axis) plot. The resulting graph yielded a straight line which is shown in Figure 6 along with the straight‐line equation. The synthesized TSP from *B. Japonica*, *O. Sayana*, and *C.bermudensis* crystallites have diameters of 126.04, 138.65, and 115.54 nm, with stress values of 2×10^7^, 4×10^7^, and 2×10^7^ N/m^2^, respectively (Table 4).

### Uniform Deformation Energy Density Model (UDEDM)

The UDM as well as USDM models explain that strain and stress is uniform throughout the crystal respectively. But no such crystal exists where both or any of these parameters are distributed uniformly throughout the crystal lattice. This is because the crystals exhibit different types of defects inside the crystal structure. Thus, a more feasible model is developed, which excludes the drawbacks of the previous two models, known as UDEDM. This model assumes a uniform energy density (μ) in the crystal lattice. This energy density is co‐related with stress and strain which can be mathematically expressed by Hook's law related to energy density shown in equation (25) and equation 26.[^50^, ^51^]

(25) $$\mu =\epsilon^{2}\;\frac{Y_{hkl}}{2}$$

(26) $$\epsilon =\sqrt{\frac{2\;\;\mu }{Y_{hkl}}}\;$$

Replacing the value of strain in equation (21), equation (27) is derived. This newly formed equation is the mathematical expression of UDEDM model.[^31^, ^50^, ^52^, ^53^]

(27) $$\beta_{total}\cos\theta =\;\frac{K_{B}\lambda }{D_{w}\;}+4sin\theta \;\frac{\sqrt{2\;\;\mu }}{\sqrt{Y_{hkl}}}\;$$

**Figure 7:**
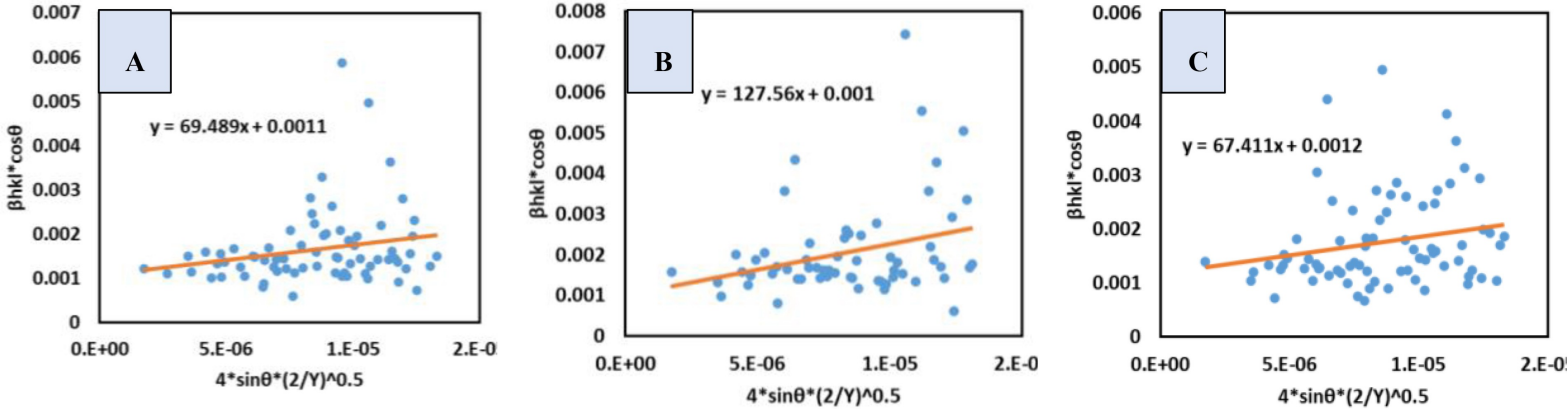
UDEDM for (A) B. japonica (B) O. sayana (C) C. bermudensis.

A straight line was drawn by putting $\beta_{total}\cos\theta$ (y‐axis) and $\;\frac{4sin\theta }{\sqrt{Y_{hkl}}}$ (x‐axis) (Figure 7). The crystal size as well as energy density were calculated by comparing the straight‐line equation with equation (27) where the slop is $\sqrt{2\;\;\mu }$ and the intercept is $\frac{K_{B}\lambda }{D_{w}\;}$ . The energy density of *B. Japonica*, *O. Sayana*, and *C. Bermudensis* was measured to be 4.82×10^3^,16.27×10^3^, and 4.54×10^3^ J/m^3^, respectively. Additionally, the dimensions of the crystallites were calculated to be 126.04, 138.65, and 115.54 nm, respectively, as shown in Table 4.

### Size‐Strain Plot Method (SSPM)

The Williamson‐Hall model predicts that line broadening is typically isotropic, implying diffracting domains are isotropic owing to micro‐strain. An average size‐strain plot (SSP) is more useful for evaluating size and strain of anisotropic crystals at lower angles. This approach is more successful in XRD examination of powdered materials since peak overlap at lower angles is less. The SSP assumes crystallite size is connected to the Lorentzian function and strain to the Gaussian function(equation 28).^[54]^

(28) $$\beta_{total}=\;\beta_{L}+\beta_{G}\;$$

The mathematical expression of the size‐strain method in shown in equation 29.[^55^, ^56^, ^57^]

(29) $${{(d}_{hkl}\beta_{hkl}\cos\theta )}^{2}=\;\frac{K_{B}\lambda }{D_{w}\;}\;\left({d_{hkl}}^{2}\beta_{hkl}\cos\theta \right)+\frac{\epsilon^{2}}{4}\;$$

**Figure 8:**
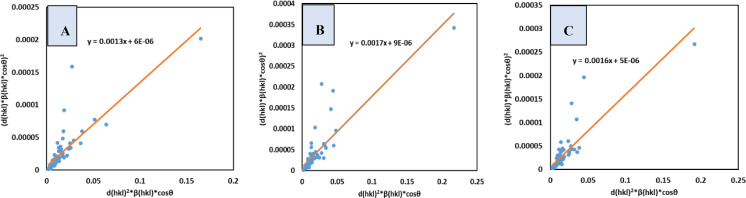
SSP for (A) B. japonica (B) O. sayana (C) C. bermudensis.

Here, ${{(d}_{hkl}\beta_{hkl}\cos\theta )}^{2}$ and $\left({d_{hkl}}^{2}\beta_{hkl}\cos\theta \right)$ were plotted along the vertical as well as x‐axis, correspondingly (Figure 8). The resulting equation equations were compared with a straight‐line equation (y=mx+c), where the slop of the equation yielded the crystallite size as well as the intercept yielded strain. The model predicted crystallite sizes of 106.65 nm for *B. Japonica*, 81.56 nm for *O. Sayana*, and 86.65 nm for *C. Bermudensis*, is shown in Table 4.

#### Halder‐Wegner Method (HWM)

The Halder‐Wegner method assumes that the XRD reflection is not solely Lorentzian or Gaussian function. Rether, it results due to the combine contribution of these two functions where the peak and tail follows the Gaussian function and Lorentzian function respectively. According to this method, the XRD peak is a vigot function. This method considered integral breadth which is based on the vigot‐function. The peak broadening using the vigot‐function can be written as equation 30.^[27]^

(30) $${\beta_{hkl}}^{2}=\;\beta_{L}\;\beta_{hkl}+{\beta_{G}}^{2}\;$$

The formula of Halder‐Wegner method is shown in equation 31.[^34^, ^58^]
(31)

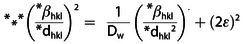




**Figure 9:**
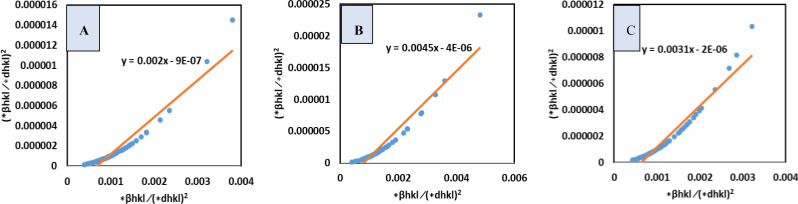
HWM for ((A) B. japonica (B) O. sayana (C) C. bermudensis.

Here,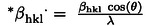 and 
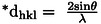
 , which denotes to integral breadth and lattice plane spacing respectively. The term 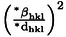 was plotted along y‐axis and 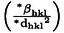 was plotted along x‐axis (Figure 9). The equation obtained from this plot was compared with a straight‐line equation. The model predicted crystallite sizes for *B. Japonica*, *O. Sayana*, and *C. Bermudensis*, with *B. Japonica* having a size of 50 nm, *O. Sayana* having a size of 22.22 nm, and *C. Bermudensis* having a size of 32.25 nm.

#### Sahadat‐Scherrer Model (SSM)

SSM is a newly developed model derived from the Scherrer model that eliminates the limitations of the other relevant models to a significant extent. The equation of this model is expressed as equation 32.[^34^, ^59^, ^60^, ^61^]

(32) $$cos\theta =\;\frac{K\lambda }{D_{S-S}}\times \frac{1}{FWHM}$$

**Figure 10:**
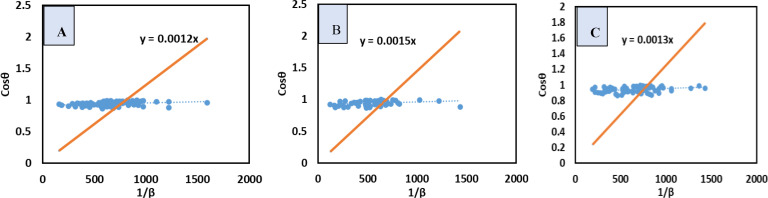
SSM for (A) B. japonica (B) O. sayana (C) C. bermudensis.

A graph was plotted according to this model putting the values of cosθ (x‐axis) and the adjacent values of $\frac{1}{FWHM}$ (y‐axis) (Figure 10). Here all the peaks of the synthesized TSP sample were considered. A straight line was generated from the plot. Another straight‐line was drawn, which is passing through the origin of the graph. The former line is the significant feature of this model, which was considered to estimate the crystallite dimension more precisely. The synthesized TSP from marine mollusks like *B. Japonica*, *O. Sayana*, and *C. Bermudensis* was measured to have crystallite dimensions of 15.54, 92.43, and 106.65 nm, respectively.

### Functional Group Analysis

**Figure 11:**
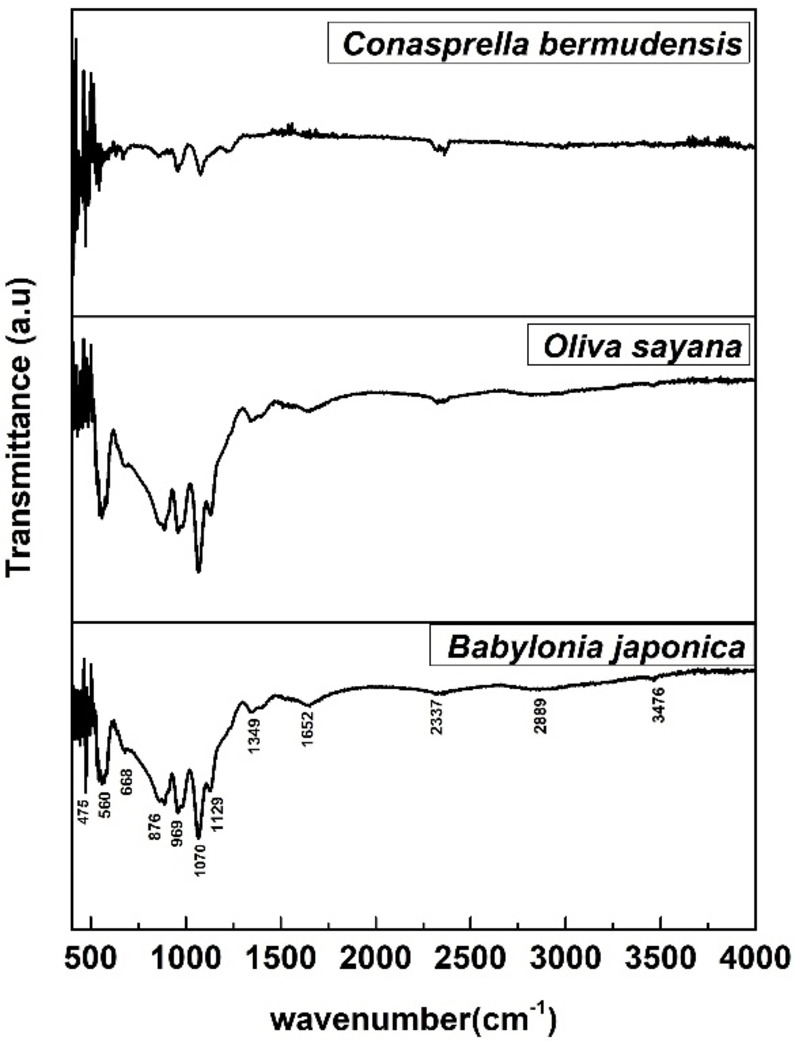
Functional group analysis for TSP.

The Fourier transform infrared (FTIR) spectrum is used to identify material compounds by identifying chemical functional groups within their molecules. The target compound, Ca(H_2_PO_4_)_2_⋅H_2_O, is commonly referred to as TSP in the fertilizer industry and MCPM in academia. Its structure includes CaO, the^[H2PO4] −^ ion, and the H_2_O molecule. The FTIR spectrum helps recognize each spectrum of the produced samples based on the basic vibrational modes of these compounds (Figure 11). The study found bending oscillations of O−P‐O groups in the range of 450–600 cm^−1^, with stretching vibrations peaks in 900–1100 cm^−1^. Out‐of‐plane as well as In‐plane oscillations of P−O bonds were observed at 800–900 cm^−1^ and 1200–1250 cm^−1^, respectively. Water molecules′ bending as well as stretching modes were observed in the ranges of 800, 2800, 3200, and 3500 cm ^1^.[^19^, ^62^]

### SEM Analysis

**Figure 12:**
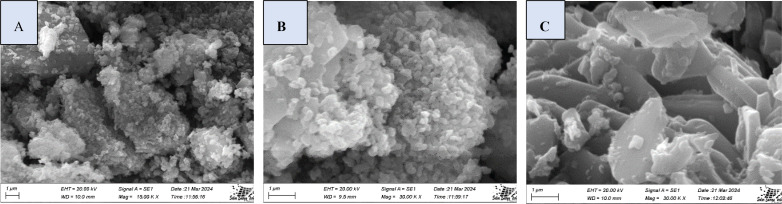
SEM images of (A) *B. japonica* (B) *O. sayana* (C) *C. bermudensis*.

The surface morphologies of synthesized TSP, obtained from marine mollusks (*B. japonica*, and *O. sayana*), reveal numerous distinct types of particles clustering on the surface. These particles are larger, more diversified agglomerates, with most being larger than a hundred nanometers in a conspicuous shape Figure 12(a–b). The synthesized TSP from *C. bermudensis* exhibits a variety of polyhedral sheet‐like pieces (Figure 12(c)) with varying particle sizes.

### EDX Analysis

**Figure 13:**
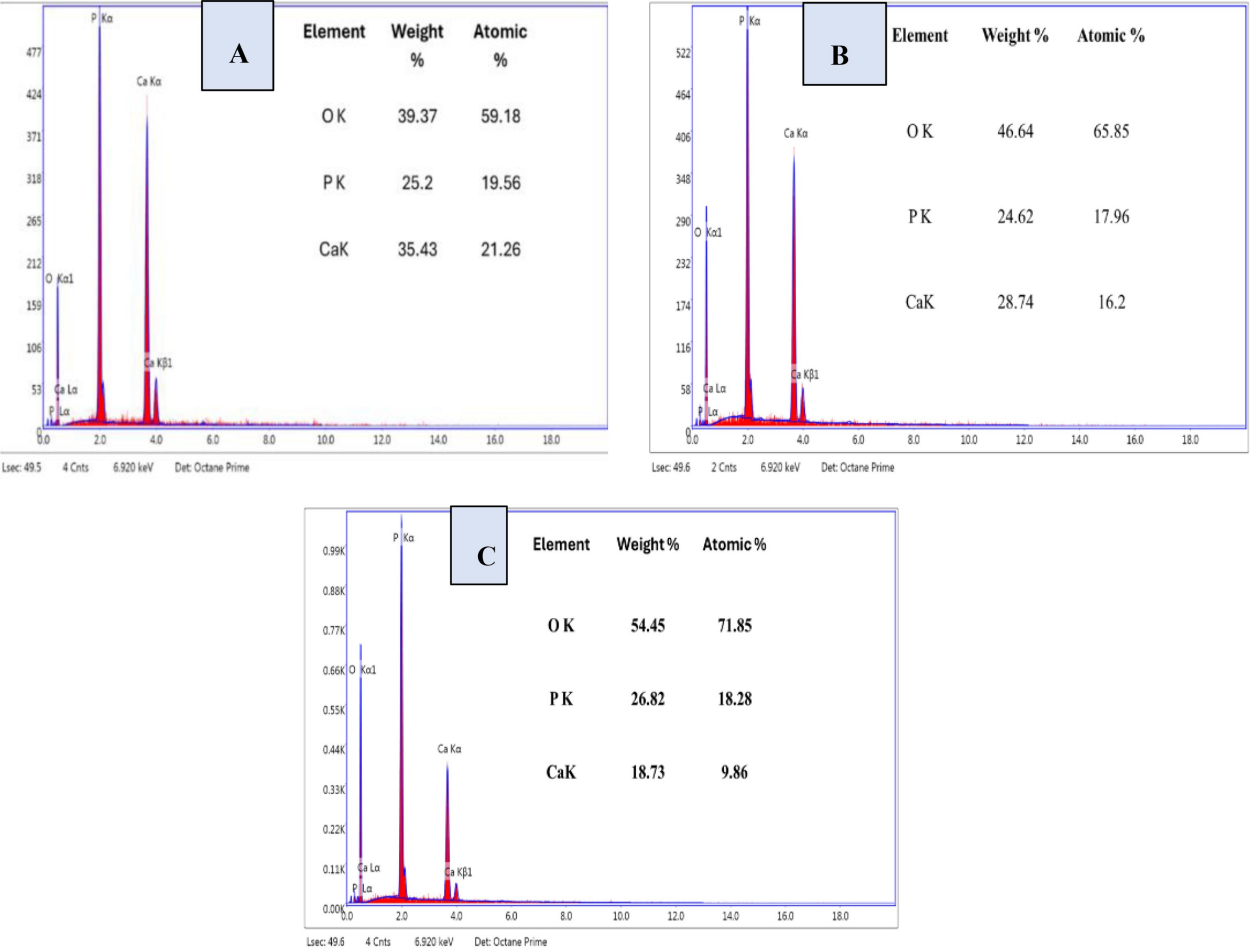
EDX analysis for Synthesized TSP(A) *B. japonica* (B) *O. sayana* .(C) *C. bermudensis*

The EDX spectrum of synthetic TSP revealed (Figure 13) varying levels of calcium, phosphate, and oxygen content among different species. *B. Japonica* had 21.26 % calcium, 19.56 % phosphate, and 59.18 % oxygen, while *O. Sayana* had 16.2 % calcium, 17.19 % phosphate, and 65.85 % oxygen. Furthermore, *C. bermudensis* had 9.86 % calcium, 18.28 % phosphate, and 71.85 % oxygen, correspondingly.

## Conclusions

The synthesis and characterization of nano‐crystallite triple superphosphate (TSP) from marine mollusk waste, including *Babylonia japonica*, *Oliva sayana*, and *Conasprella bermudensis*, presents a promising alternative to traditional phosphate rock ore production methods. The study found that using 60 % (w/w) phosphoric acid as phosphate source does not necessitate a further drying step. The generated TSP was analyzed using various characterization techniques, which includes TGA, XRD, EDX, FT‐IR, as well as SEM. The TGA data indicates that water eliminating limits can be considered during the drying of typical P‐fertilizer produced in the industry. The Williamson‐hall model is regarded as the most accurate in calculating the crystallite dimension, stress, strain, and energy density values, despite the uniformity in measurements across all models, although the existence of identical crystallite sizes. The study on synthesizing nano‐crystallite TSP from marine mollusk waste has limitations, including being limited to three waste species and not optimizing synthesis conditions. It also lacks detailed characterization of the nano‐crystallite structure and a comprehensive life cycle assessment to quantify environmental benefits. Future research should explore a wider range of waste sources and investigate potential applications beyond fertilizers.

## Author Contributions

Md. Kawsar synthesized and characterized the gypsum, executed the experiment, and wrote the draft and original manuscript. Md. Sahadat Hossain conceived and designed the experiment and analysed the data. Sumiya Akter assisted in collecting data along with Md. Kawsar. Md. Farhad Ali done the TGA experiment. Newaz Mohammed Bahadur and Samina Ahmed supervised the findings of this work. Samina Ahmed supervised the overall work and managed the required facilities.

## Conflict of Interests

There are no conflicts to declare.

1

## Data Availability

Data will be made available on request from authors.
